# Medical physics aspects of cancer care in the Asia Pacific region

**DOI:** 10.2349/biij.4.3.e33

**Published:** 2008-07-01

**Authors:** T Kron, KY Cheung, J Dai, P Ravindran, D Soejoko, K Inamura, JY Song, L Bold, R Srivastava, L Rodriguez, TJ Wong, A Kumara, CC Lee, A Krisanachinda, XC Nguyen, KH Ng

**Affiliations:** 1 Physical Sciences, Peter MacCallum Cancer Centre, and RMIT University, Melbourne, Australia; 2 Department of Clinical Oncology, Prince of Wales Hospital, Hong Kong, China; 3 Cancer Institute (Hospital), Chinese Academy of Medical Sciences, China; 4 Department of Radiation Oncology, Christian Medical College, Vellore, India; 5 Physics Department, University of Indonesia, Jakarta, Indonesia; 6 Dept of Radiology & Medical Engineering, Kansai University of International Studies, Hyogo, Japan.; 7 Department of Radiation Oncology, Chonnam National University Hospital, Republic of Korea; 8 Radiotherapy Department, National Cancer Center, Ulaanbaatar, Mongolia; 9 B.P.Koirala Memorial Cancer Hospital, Bharatpur, Chitwan, Nepal; 10 Department of Radiation Oncology, Jose R. Reyes Memorial Medical Center, Manila, Philippines; 11 Department of Therapeutic Radiology, National Cancer Centre, Singapore.; 12 Division of Medical Physics, National Cancer Institute, Sri Lanka; 13 Department of Medical Imaging and Radiological Sciences, Chang Gung University, Taiwan; 14 Department of Radiology, Faculty of Medicine, Chulalongkorn University, Bangkok, Thailand; 15 K Hospital, National Cancer Institute, Hanoi, Vietnam; 16 Department of Biomedical Imaging, University of Malaya, and Medical Physics Unit, University of Malaya Medical Centre, Kuala Lumpur, Malaysia

**Keywords:** Medical physics, education

## Abstract

Medical physics plays an essential role in modern medicine. This is particularly evident in cancer care where medical physicists are involved in radiotherapy treatment planning and quality assurance as well as in imaging and radiation protection. Due to the large variety of tasks and interests, medical physics is often subdivided into specialties such as radiology, nuclear medicine and radiation oncology medical physics. However, even within their specialty, the role of radiation oncology medical physicists (ROMPs) is diverse and varies between different societies. Therefore, a questionnaire was sent to leading medical physicists in most countries/areas in the Asia/Pacific region to determine the education, role and status of medical physicists.

Answers were received from 17 countries/areas representing nearly 2800 radiation oncology medical physicists. There was general agreement that medical physicists should have both academic (typically at MSc level) and clinical (typically at least 2 years) training. ROMPs spent most of their time working in radiotherapy treatment planning (average 17 hours per week); however radiation protection and engineering tasks were also common. Typically, only physicists in large centres are involved in research and teaching. Most respondents thought that the workload of physicists was high, with more than 500 patients per year per physicist, less than one ROMP per two oncologists being the norm, and on average, one megavoltage treatment unit per medical physicist.

There was also a clear indication of increased complexity of technology in the region with many countries/areas reporting to have installed helical tomotherapy, IMRT (Intensity Modulated Radiation Therapy), IGRT (Image Guided Radiation Therapy), Gamma-knife and Cyber-knife units. This and the continued workload from brachytherapy will require growing expertise and numbers in the medical physics workforce. Addressing these needs will be an important challenge for the future.

## INTRODUCTION

Medical Physics is an applied branch of physics which is concerned with the applications of physics concepts and methods to the diagnosis and treatment of human disease (compare, for example: http://www.aapm.org/medical_physicist/default.asp). As this is a vast field, it is common to divide medical physics into subspecialty areas such as radiology, nuclear medicine and radiation oncology medical physics. In radiation oncology, medical physicists are accepted as important members of the team delivering radiation therapy. They work with oncologists, radiation therapists (also referred to as RTTs, technologists or therapy radiographers), nurses and engineers to provide quality care for cancer patients. In addition to this, they provide services to other medical professions such as radiologists and nuclear medicine specialists, whose input into cancer care is essential.

The Asia-Oceania Federation of Organizations for Medical Physics (AFOMP) was founded in 2000 to promote medical physics in the Asia and Oceania region and the advancement in status and standard of practice of the medical physics profession (http://www.afomp.org/). It states that:

“A qualified Medical Physicist is a person who possesses a university degree of at least a master level or equivalent in physical science or engineering science and works in alliance with medical staff in hospitals, universities or research institutes. He/she shall also have received clinical training in the concepts and techniques of applying physics in medicine, including training in the medical application of both ionizing and non-ionizing radiation. This person shall have a thorough knowledge and be able to practice independently in one or more sub-fields of medical physics, including imaging physics, radiation therapy physics, nuclear medicine physics and radiation protection.”

This definition is similar to many others that have been proposed by organisations all around the world such as the European Federation of Organisations for Medical Physics (EFOMP – http://www.efomp.org/) [1] or the American Association of Physicists in Medicine (AAPM – www.aapm.org). For example, the International Atomic Energy Agency (IAEA) specified recently in its TecDoc 1296 that:

“Medical physicists practicing in radiotherapy (or radiation oncology) must be qualified as physicists with academic studies in medical physics (typically at postgraduate level) and clinical training in radiotherapy physics. Medical physicists specialized in radiotherapy physics will be referred to as clinically qualified radiotherapy physicists.” [2]

The important points common to all these definitions are that physicists working in a radiation oncology department have both an academic education and clinical training. However, anecdotal evidence shows that there is a wide variety of standards and requirements for medical physicists worldwide.

It is therefore timely to explore how medical physics is practised in the different countries/areas of the Asia Oceania region. It is the aim of the present article to

- Provide general information on the tasks undertaken by medical physicists in the region
- Document what education and practical experience is required to become a medical physicist
- Explore resources, status and job satisfaction of medical physicists

While this would apply similarly to nuclear medicine and radiology, the present work focuses on radiotherapy and radiation oncology medical physicists (ROMPs).

## METHODS

A simple questionnaire was designed to determine education levels, work patterns and status of medical physics in radiation oncology. The questionnaire was sent to 20 eminent physicists in the region who have been active in the field for several years. Many of them have represented their medical physics organizations at AFOMP, the International Organization of Medical Physics (IOMP) and IAEA and, as such, were considered to be familiar with the state of medical physics in their respective countries.

The questionnaire was distributed in English and covered the following fields:

### A. Education

1. What is the typical education level for physicists working in radiation oncology?
2. Are these levels or similar education opportunities available in your country?
3. What type of opportunities are there for medical physicists to participate in continuing professional development (CPD)?

### B. Staffing

1. What is the total number of radiotherapy physicists in your country?
2. What is the number of megavoltage external beam radiotherapy units in your country? (Please list Cobalt and linear accelerators separately)
3. What is the ratio of ROMPs relative to the number of oncologists?
4. What is the ratio of ROMPs relative to the number of patients treated per annum?

### C. Typical time spent on specified tasks for ROMPs (hours per week)?

### D. Professional organisations

1. Name of your local professional organisation(s)
2. How many members does this organisation have?
3. Are ROMPs members of other professional organisations in your country? (examples: radiation oncologists, radiology)
4. Are ROMPs members of overseas professional medical physics organisations? (examples: IPEM, AAPM)

### E. What resources are typically available for ROMP work in your country?

1. Dosimetry and QA equipment
2. Are reference literature and books available?
3. Do ROMPs have generally access to the Internet?
4. Are discussions with senior colleagues possible?

### F. Research and teaching

1. Are ROMPs participating in research activities?
2. Are ROMPs participating in clinical trials?
3. Are ROMPs participating in teaching?

### G. Overall satisfaction in the areas of professional recognition, remuneration and workload.

In addition to this questionnaire, participants were invited to provide as many free form comments as necessary. The original time frame for answering the questions was 2 weeks; however, responses given after a longer period were accepted. They reflect the status of March/April 2008. On some occasions, additional details were elicited and provided in communication with participants.

## RESULTS

Answers were received from 17 countries/areas representing more than 2800 radiation oncology medical physicists. This constitutes a response rate of 80%. Many of the answers were received within a few days of sending out the questionnaire. Tables 1 to 5 show the results.

**Table 1:** Education, training and continued professional development (CPD) of medical physicists

****	**Academic training for ROMPs(expected degree)**	**Typical length of clinical training(years)**	**Available locally(Y/N)**	**Opportunities for CPD**
Australia	MSc	3	Y	conference every 2 years, in-house lectures, ACPSEM organises local seminars
Hong Kong China	MSc	2	research MSc	large variety, in general good access
India	MSc, also Dip RP from BARC* well established	1 (2 year residency program is also established at some institutions)	Y	excellent in university hospitals, not as good elsewhere
Indonesia	BSc, MSc preferred	2 (planned)	Y	generally no easy access as too few senior staff
Japan	BSc (a higher degree reduces the time required for clinical training)	2 to 7 years	Y (based on ‘radiation technology)	in-house lectures, research activity required for re-certification
South Korea	MSc	3	Y	lectures managed by KSMP
Malaysia	BSc, MSc preferred		Y	in-house lectures, other funding through MedPhys centres
Mongolia	none established		N	
Nepal	MSc	1	N	few in-home institutes
New Zealand	MSc	3	Y	
Philippines	MSc preferred	2 (planned)	Y	some local activities, conference funding difficult
PR China	MSc preferred		Y	short time attachments overseas, in-house lectures and meetings/workshops
Singapore	MSc preferred	2 + overseas attachment	limited after BSc	wide variety with good access to funding for overseas conferences
Sri Lanka	MSc to be completed within 5 years after selection		MSc in Medical Physics	Funding for conference
Republic of China (Taiwan)	BSc, MSc preferred		Y	Variety, often organised by CSMPT
Thailand	MSc		Y	many opportunities
Vietnam	BSc, MSc preferred		BSc training	in-house lectures, funding for conferences possible

* BARC = Bhabha Atomic Research Centre, Mumbai

**Table 2:** Equipment, staffing and resources – abbreviations: CK = cyberknife, GK = gammaknife, HT = helical tomotherapy, P = proton (and heavy ion) radiotherapy, SRS = stereotactic radiosurgery, MT = microtron

	**ROMPS(#)**	**Brachytherapy**	**Tele Cobalt(#)**	**Linacs(#)**	**Other treatment units(#)**	**Oncologists/ ROMP(ratio)**	**Patients/ ROMP(ratio)**	**MV Machine/ ROMP(ratio)**	**Population 2008, US Census Bureau**	**MV Machine/ Mn Pop(ratio)**
Australia	224 (3/4 experienced)	offered by about half the centres	0	120	IMRT, IGRT, SRS	1.5	300	0.54	20.6Mn	5.83
Hong Kong China	42	full range	0	30	HT 1, CK 1, GK 1	2.0	400	0.79	7.0Mn	4.71
India	550		283	104	HT 1, GK 5, IMRT, IGRT	2.0	300 to 400	0.70	1148Mn	0.34
Indonesia	38	13 brachy-therapy units	14	15		1.0	290	0.76	237.5Mn	0.13
Japan	383*		0	889	HT 5, P 2, MT 14			2.32	127.3Mn	7.0
Korea	66		2	100	HT 7, CK 5, P 1	2.1	482.5	1.55	49.2Mn	2.07
Malaysia	60	10 brachy-therapy units	1	30	CK 1, IMRT, IGRT	0.5	300	0.53	25.3Mn	1.26
Mongolia	3		2			2.5	500	0.67	3.0Mn	0.67
Nepal	10		4	3		1.5	400	0.70	29.5Mn	0.24
New Zealand	44	in few centres	0	30	IMRT, IGRT			0.68	4.2Mn	7.1
Philippines	30	16 centres with brachytherapy	9	20	GK 1	3 to 4	800	1.00	92.7Mn	0.32
PR China	1181	400 brachy-therapy units	472	918	GK 149 + SRS and IMRT	5.0	400	1.18	1330Mn	1.04
Singapore	13	full range	0	17	HT1, GK 1	2.5	500	1.38	4.6Mn	3.91
Sri Lanka	8	large workload with 131-I	10	1		2.5	2000	1.38	21.1Mn	0.52
Republic of China (Taiwan)	100 (60 certified)		4	100	HT 6, CK 4	2.0	250	1.14	22.9Mn	5
Thailand	76		22	37		1 to 2	500	0.78	65.5Mn	0.9
Vietnam	25		15	8		3.0	800	0.92	86.1Mn	0.27
**Total**	2853*	****	**838**	**2422**	**mean**	**2.2**	**566**	**1.0**	****	**2.43**

* not specific to radiation oncology – this includes all medical physicists;

# the questionnaire did not specifically ask for IMRT and IGRT capable units. It was listed here where the respondents indicated its availability. As such this is not a full listing of equipment with these capabilities; however, it indicates the complexity of at least some of the equipment available in the Asia Pacific region.

**Table 3:** Typical work patterns

	**Radiotherapy Treatment Planning(hours per week)**	**Radiation Protection(hours per week)**	**Research(hours per week)**	**Teaching(hours per week)**	**Engineering/ maintenance(hours per week)**	**Official working hours(hours per week)**
Australia	6 (checks, IMRT and brachy)	2	varies - in few centres research positions available	2	2	38
Hong Kong China	15	2	active involvement	4	3	44
India	8	4	4 in major institutions	4	8	
Indonesia	20	2	only in centres involved with education (variations)	2 – 4 (performed at centres involved with education)	2	40
Japan			Medical physicists are involved	Medical physicists are involved		
Korea	15	2	4	2	2	
Malaysia	15	2		3		
Mongolia	15	5	0	5	2.5	35
Nepal	25 to 30	2 to 10		5 (not all involved)		42
New Zealand	6 (checks)	2	4	2	2	38
Philippines	25 to 30	2	little	1	insignificant	40
PR China	25	1	varies	varies	6	40 to 50
Singapore	15	1	1	1		44
Sri Lanka	25	10	0	5		
Republic of China (Taiwan)	24	1	2	2		
Thailand	10	3	5 in uni hospital	5 in uni hospital	2	
Vietnam	18	1	2	1	4	42 (six days/week)
**mean**	**17.1**	**2.7**				

**Table 4:** Professional organisations and professional resources available

****	**Professional organisation**	****	**Dosimetry equipment**	Access to*	****	****
****	**Name (see appendix of acronyms)**	**No. members**	availability*	**literature**	**internet**	**colleagues**
Australia	ACPSEM	500^&^	e	e	e	g
Hong Kong China	HKAMP	72	e	e	e	g
India	AMPI and brachysociety	1475	g	a	g	g
Indonesia	IKAFMI	40	g	a	a	a
Japan	JSMP, JRS – none specific for ROMPs					
Korea	KSMP	350	e	e	e	g
Malaysia	IFM /MP subgroup, MAMP	30	a	g	g	g
Mongolia	some members of RTT		a	a	g	g
Nepal	AMPN	10	a	n	n	n
New Zealand	ACPSEM	500^&^				
Philippines	POMP	70	adequate in top centres not in government centres	n	g	g
PR China	CSMP (most also member of CSRO)	1500	a (some centres e)	limited	g	only in 25% of centres
Singapore	SMP (Society of MP)	11	e	e	e	g
Sri Lanka			a	n	g	n
Republic of China (Taiwan)	CSMPT	250	e	e	e	e
Thailand	TMPS	90	uni: a; others often n	g	g	g
Vietnam	several - VAMP to be established soon	120?	n	n	n	g

& includes Australian and New Zealand members * categories: excellent: e, good: g, acceptable: a, not adequate: n

**Table 5:** Satisfaction on a scale of 1 (worst) to 5 (best) - ) – please note that the satisfactory ratings are independently estimated by the individual authors without making reference to or comparison with other countries’ norms. The ratings indicated by the authors are estimated based on different standards or norms and therefore have no correlations.

****	**Professional recognition**	**Remuneration**	**Workload(1 = too much,5 = easy)**	**Overall**	**Important comments**	****
Australia	3	3	2	3	variation in states	significant improvement over last years
Hong Kong China	3	4	3	3*	most ROMPs work significantly over time	
India			increasing	3	high tech has improved status	very significant variations in salary
Indonesia	3	1	2 - 3	3	formal recognition in 2007	research and teaching is not established yet internally
Japan					many recent medical physicists are radiological technologists by training	since 2007 development of education and training for ROMPs
Korea	3	3	2	3*		
Malaysia	2	3	3	3*	professional recognition needs to be improved	
Mongolia				3 (if developed country is 5)		
Nepal	2	3	3	2*	medical physics is new and as such recognition not good	not an IAEA member
Philippines	3.5	3.5	2 (many are overworked)	3*	difference between public and private facilities	MPs also represented in government agencies
PR China				2	status has improved with 3D CRT and IMRT	no professional title as yet - hinders promotion
Singapore	3.5	3.5	3.5	3.5*	senior MPs have significant administrative duties	
Sri Lanka	2	2	1	2*		
Republic of China (Taiwan)	4	3	2	3*		
Thailand	4	4	3	3.5*		
Vietnam	2	2	3	3	VAMP to be established in 2008	outside assistance needed

* estimate by the author

About half of the respondents provided additional information in free form (up to several pages). This information was included in the tables wherever possible. This has resulted in some columns that list data not explicitly covered in the questionnaire (eg brachytherapy and other treatment units). The information in these areas must be seen as preliminary only.

## DISCUSSION

The fast and comprehensive reply of respondents in most countries/areas illustrates the importance ROMPs place on documentation of their practice and collaboration within the Asia Oceania region.

### Education and training

All respondents agreed on the need for academic education and clinical training. This is very much in line with the definitions of medical physics listed in the introduction and the thinking in North America (http://www.aapm.org) and Europe (http://www.estro.be and http://www.efomp.org/ ) [3]. It is interesting to see in table 1 that most respondents see the need for a higher degree as an entry requirement for the profession. Without doubt, this reflects the increasing complexity of the field. Most medical physics programs throughout the world are postgraduate programs that provide specialist knowledge on top of basic skills in physical sciences and mathematics (compare eg http://www.campep.org/ or http://www-naweb.iaea.org/nahu/dmrp/syllabus.shtm). Access to relevant courses and university training appears to be available in most countries/areas in the region.

More complicated is the issue of clinical training. Again, virtually all respondents agreed that clinical training should be required prior to being able to practise radiation oncology medical physics. The typical time period required for this varied between 1 and 3 years. However, while a structured clinical training program is deemed to be essential, it is only available in a few countries/areas. One can speculate about the reasons; however, low staff number and high workload of experienced clinical physicists appear to be contributing factors that make it difficult for practising ROMPs to dedicate time for teaching and research. As the questionnaire shows, teaching and research is often only part of the job description for physicists in large academic centres. It also needs to be noted that a lot of the teaching hours listed in table 3 are directed to other professions such as doctors in training.

In any case, the desired education for ROMPs will require between six and eight years after finishing secondary school – a significant time commitment that may not be reflected in salaries and status in all cases.

Given the rapid advances and changes in technology and the need to work with potentially hazardous equipment, continuing professional education appears to be essential. It is difficult to compare the answers in table 1 – however, it is clear that there is no uniform access to relevant education within the region. A more specific questionnaire would be required to determine more precisely the perceived needs and available training and educational offerings. As most medical physicists reported good access to the Internet (table 4), there is an opportunity to provide online resources for continuing professional development (CPD).

It is also important to note that CPD is an integral part of certification of professionals [4]. As professional responsibility increases and societies expect high professional standards, certification of ROMPs will become necessary in all countries/areas. Access to adequate education, clinical training and CPD are essential for this to happen.

### Resources and staffing

The percentage of cancer patients who have access to radiotherapy services varies widely throughout the world as illustrated recently for South America [5]. The number of megavoltage treatment units also varies significantly amongst countries/areas in the region, as can be seen in table 2. Interestingly the number of ROMPs per machine and per oncologist is fairly uniform in all countries/areas. This illustrates that employers and health systems see physicists as a support person for other staff and equipment rather than as a direct contributor to patient treatment. As such, it is not surprising that the number of patients per physicist varies more significantly (250 to 800) than the number of physicists per machine. Given the fact that physics tasks are increasingly linked to the number of patients treated (eg treatment planning, patient specific QA) this may further disadvantage physicists in countries/areas with few megavoltage machines.

It is encouraging to see that most countries/areas have a professional association that represents medical physicists. This provides an important framework that can be used for promotion of medical physics issues and patient safety, as well as education and sharing of resources.

### Typical tasks and workload for ROMPs

Apart from Australia and New Zealand, physicists in other countries/areas spent most of their time on radiotherapy treatment planning. This is a significant responsibility that combines optimisation of treatment approaches for individual patients with developing planning methods and commissioning of treatment planning systems [6,7]. The emphasis on treatment planning reflects a patient and service focus in the employment of most medical physicists. Unfortunately, the workload and service focus result in only a few medical physicists being actively involved in teaching and research. Both activities have the potential to enhance job satisfaction and profile of staff – more importantly, they would contribute to the much-needed clinical training required to ensure adequate supply of qualified ROMPs in the future.

There is no doubt that, due to increasing awareness of radiation safety and accident prevention [8-10], the responsibility of physicists is increasing. ROMPs in all countries/areas spent at least part of their time in radiation protection. However, it is the advances in technology and the introduction of computing and imaging in radiotherapy, that makes the role of physicists more and more important. All these advances make quality assurance and accurate dosimetry increasingly important. However, it is interesting to note that while dosimetric protocols have improved and simplified [11,12], most protocols for quality assurance do not yet include guidance for advanced technology [13,14]. This demonstrates the need for independent critical thinking and a high level of professional competence for medical physicists in order to develop procedures appropriate for their respective institution.

Medical physicists typically have expertise in many different areas such as radiation dosimetry, radiation protection and medical imaging. Maybe not surprisingly, Weibo Yin reported recently that the largest percentage growth of staff numbers in radiation oncology in mainland China from 1997 to 2006 was in medical physics [15]. Given the fact that diagnostic procedures are increasingly important in detecting and outlining cancer, the role of medical physicists in imaging will increase.

Not listed in the tables is the involvement of medical physicists in clinical trials. In several countries/areas this was noted with a typical time allocation of a few hours. It appears that clinical trials will continue to be essential in defining best clinical practice and there is a trend to extend this to more countries/areas [16]. It can be expected that medical physics involvement in these trials for quality assurance and resource allocation is also likely to continue to grow in the future.

### Status and job satisfaction

Most respondents felt that medical physicists have reasonable professional recognition. More significantly, several of those who responded indicated an improvement in professional recognition and status over time. This is no doubt related to the more visible need for scientific support for complex treatment using sophisticated equipment. Remuneration was found to vary largely between countries/areas and even within some countries such as India. However, at least in academic and private institutions, there appears to be an acceptable level of salaries. It may be of concern that in times of staff shortages these centres will attract most of the qualified staff while smaller public centres with a focus on service provision may find it hard to attract and maintain staff.

It is also interesting to note that many of the respondents stated that ROMPs, in general, work long hours. From the data it is impossible to tell if this is adequately remunerated – however, it is clear that there are not enough trained medical physicists to perform the increasing number of tasks. This is not confined to the region but appears to be a worldwide phenomenon [17].

### Limitations of the study

The present work has several limitations. They pertain to the need to use a relatively brief questionnaire with many important omissions, such as brachytherapy or the details on quality assurance and the way physicists interact with colleagues. This is an aspect that will be addressed in future surveys. Another significant problem is the impossibility of characterising widely varying practices and employment conditions in many countries/areas with a single answer. When salaries, eg in India, vary by more than a factor of 10 between different employers, it is difficult to derive a single number, eg for job satisfaction.

The manuscript provides only a snapshot of conditions for ROMPs in most countries/areas in the Asia/Pacific Region. The time frame for participants to respond to the questionnaire did not allow them to perform detailed research. Some countries/areas had data readily available due to other recent activities [15,18]. However, in others, the results reflect a considered judgement of the participant.

The manuscript is based on a simple questionnaire that is only aimed at determining the broad picture. There may be bias in the selection of the participants and others may have provided answers with different emphasis. In addition to this, the questionnaire was only distributed in English which could result in differences in interpretation as English is not the first (or even the second) language in many participating countries/areas.

## CONCLUSION

Given the variability of the situation of ROMPs throughout the region it is surprising how similar many of the answers were. This illustrates that medical physicists share a common work environment and face similar challenges independent of the country they are working in. This forms the foundation for effective communication in larger organisations such as AFOMP. However, significant differences in resources remain and it will make sense to pool information and resources wherever possible. Organisations such as AFOMP have an important role to play by defining professional responsibilities, and educational standards. An even more important role is to bring physicists together by organising conferences and workshops. Given the fact that many physicists work in small centres in isolation, this is essential for safe and effective use of equipment for cancer treatment. Finally, journals such as biij are essential in disseminating information – more so as it is an open-access journal.

The present survey provides only a snapshot in time. It will be essential to repeat this type of regional study to map trends in medical physics employment and provide longitudinal data essential for long-term planning of workforce and training development for medical physicists in the Asia/Pacific Region.

## Acronyms for professional associations:

- AAPM: American Association of Physicists in Medicine
- ACPSEM: Australasian College of Physicists and Engineers in Medicine
- AFOMP: Asia-Oceania Federation of Organizations for Medical Physics
- AMPI: Association of Medical Physicists in India
- AMPN: Association of Medical Physicists of Nepal
- CSMP: Chinese Society of Medical Physics
- CSMPT: Chinese Society of Medical Physics, Taipei
- CSRO: Chinese Society of Radiation Oncology
- HKAMP: Hong Kong Association of Medical Physics
- IFM: Institut Fizik Malaysia
- IKAFMI: Indonesian Medical Physics Association
- JRS: Japan Radiological Society
- JSMP: Japan Society of Medical Physics
- KSMP: Korean Society of Medical Physics
- MAMP: Malaysian Association of Medical Physics
- POMP: Philippine Organization of Medical Physicists
- SMP: Society of Medical Physicists Singapore
- TMPS: Thai Medical Physicists Society
- VAMP: Vietnam Association for Medical Physicists
